# Updated Toolbox for Assessing Neuronal Network Reconstruction after Cell Therapy

**DOI:** 10.3390/bioengineering11050487

**Published:** 2024-05-14

**Authors:** Ana Gonzalez-Ramos, Claudia Puigsasllosas-Pastor, Ainhoa Arcas-Marquez, Daniel Tornero

**Affiliations:** 1 Stanley Center for Psychiatric Research at Broad Institute of MIT and Harvard, Cambridge, MA 02142, USA; 2 Laboratory of Neural Stem Cells and Brain Damage, Department of Biomedical Sciences, Institute of Neurosciences, University of Barcelona, 08036 Barcelona, Spain; 3 Institut d’Investigacions Biomèdiques August Pi i Sunyer (IDIBAPS), 08036 Barcelona, Spain; 4 Centro de Investigación Biomédica en Red sobre Enfermedades Neurodegenerativas (CIBERNED), 28029 Madrid, Spain

**Keywords:** cell therapy, functional connectivity, optogenetics, chemogenetics, DREADD, calcium imaging, indicator, sensor, monosynaptic tracing, rabies virus

## Abstract

Cell therapy has proven to be a promising treatment for a range of neurological disorders, including Parkinson Disease, drug-resistant epilepsy, and stroke, by restoring function after brain damage. Nevertheless, evaluating the true effectiveness of these therapeutic interventions requires a deep understanding of the functional integration of grafted cells into existing neural networks. This review explores a powerful arsenal of molecular techniques revolutionizing our ability to unveil functional integration of grafted cells within the host brain. From precise manipulation of neuronal activity to pinpoint the functional contribution of transplanted cells by using opto- and chemo-genetics, to real-time monitoring of neuronal dynamics shedding light on functional connectivity within the reconstructed circuits by using genetically encoded (calcium) indicators *in vivo*. Finally, structural reconstruction and mapping communication pathways between grafted and host neurons can be achieved by monosynaptic tracing with viral vectors. The cutting-edge toolbox presented here holds immense promise for elucidating the impact of cell therapy on neural circuitry and guiding the development of more effective treatments for neurological disorders.

## 1. Introduction

In the quest to repair brain damage, cell therapy has emerged as a promising strategy for restoring neuronal function and connectivity. But the ultimate goal to accomplish optimum long-term recovery implies functional integration of the grafted neurons into the host neuronal circuitry [1]. However, the assessment of effectiveness of cell-based interventions in reconstructing neural networks poses significant challenges [2]. To address this, researchers have turned to a sophisticated toolbox of molecular techniques that offer unprecedented insights into the integration and function of transplanted cells within the host brain.

On one hand, optogenetics and chemogenetics allow precise manipulation of neuronal activity. By genetically engineering cells to express light-sensitive or chemically responsive proteins, researchers can selectively modulate the activity of specific neuronal populations, providing invaluable insights into the functional integration of transplanted cells [3]. Furthermore, advances in calcium imaging have revolutionized our ability to monitor neuronal activity *in vivo* with unparalleled precision [4]. Genetically encoded calcium indicators enable real-time visualization of neuronal dynamics, offering a window into the functional connectivity of neural circuits following cell therapy. Finally, monosynaptic tracing using viral vectors has become indispensable for mapping the intricate pathways of neuronal communication [5]. By selectively labeling interconnected neurons, researchers can track the integration of transplanted cells into existing neural networks, providing crucial clues about the mechanisms underlying cell therapy-induced functional recovery.

**Figure 1:**
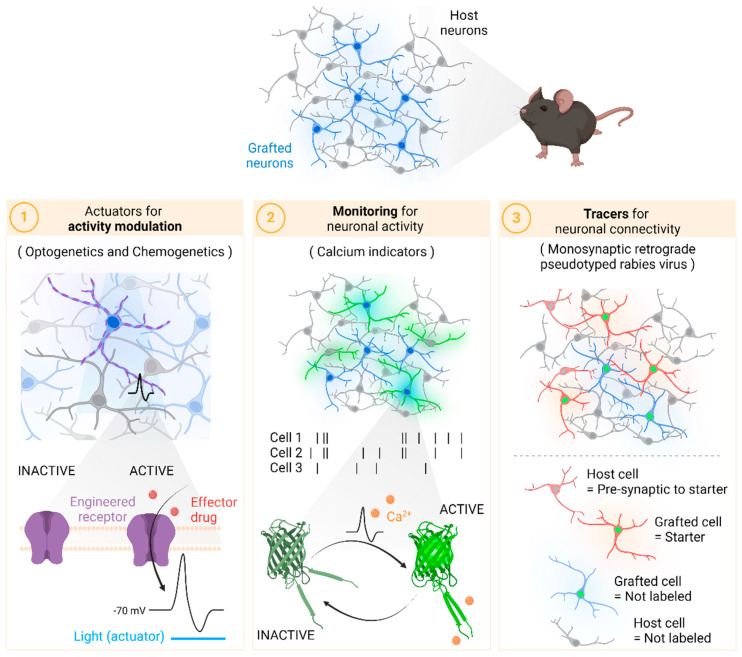
Scheme summarizing the three groups of tools for the evaluation of network reconstruction discussed throughout the review. Created with BioRender.

In this article, we review the latest advancements in molecular tools and technological applications for assessing neuronal network reconstruction after cell therapy. From optogenetics and chemogenetics to calcium imaging and viral tracing, these cutting-edge techniques hold immense promise for elucidating the impact of cell-based interventions on neural circuitry and paving the way for novel therapeutic strategies in neurological disorders (Figure 1).

## 2. Actuators for Activity Modulation

In the past decades, two new techniques have been developed granting an unprecedented efficient and noninvasive manipulation of the grafted cells and surroundings. Consequently, these techniques afford the assessment of grafted cells’ functional integration into the host brain tissue both *in vitro* and *in vivo*. These powerful molecular tools are known as optogenetics and chemogenetics and have pushed the boundaries in neuroscience by allowing activity control on targeted cells. Now, it is possible to modulate the activity of type- and region-specific neurons and other brain cells.

Both tools use actuators that physiologically do not affect neurons, albeit they do when engineered receptors are expressed in the cellular membrane. Indeed, the receptor or effector is introduced typically using viral vectors in the chosen cellular population, while the other cell populations remain intact. For instance, stem cell-derived neuronal precursors can be genetically engineered to express the effector protein prior to transplantation into the host tissue, which thus will be unaltered. Then, when the actuator is administered, light for optogenetics and small-molecule chemical compounds for chemogenetics, it will interact with the effector in the grafted cell. An alternative strategy could be to not modify the transplanted cells, but rather genetically engineer mice to express the effector protein under the control of an inducible system such as the tetracycline (Tet-off) promotor [6,7] or Cre-mediated recombination [8], and evaluate the afferent connections to the transplanted cells.

### 2.1. Optogenetics

Optogenetics emerged in 2005 when Deisseroth and colleagues demonstrated that opsin genes found in microorganisms like algae could be used safely to control cellular activity in neurons [9,10,11,12]. Channelrhodopsin-2 (ChR2) was the first light-activated channel developed and proven in mammalian cells. This channel is activated by 470 nm wavelength blue light [9] that leads to the passive diffusion of positively charged ions across the channel, resulting in the depolarization of the cell membrane potential. Other opsins generating different electrochemical responses like hyperpolarization of the membrane resulting in neuronal inhibition have been discovered, including halorhodopsins (NpHR) and archaerhodopsins (Arch), which are inward chloride pumps and outward proton pumps, respectively [10,13]. Thenceforth, various engineered versions of natural opsins have been developed with improved properties. Some of those properties are: (1) different activation wavelengths like the red-shifted peak action spectra ChrimsonR and Chronos compared to the initial blue-activated ChR2 [14,15]; (2) increased photocurrent amplitude such as C1V1; and (3) increased channel kinetics as achieved by ChETA or ChrimsonR [16,17]. Currently, it is possible to both silence (hyperpolarize) and incite (depolarize) neuronal firing in the same neuronal population by combining engineered opsins and wavelengths of light [3,15,18,19].

### 2.2. Chemogenetics

Chemogenetics had an earlier start, although with protracted years of optimization [20,21]. The earliest evidence for the development and use of a chemogenetic tool was in 1991, when Strader and colleagues mutated the β-adrenoceptor to be activated by catechol-containing esters and ketones [22]. This was followed by numerous variants based on different proteins including kinases and ligand-gated ion channels [23,24,25,26,27,28,29,30,31] until 2010, when Designer Receptors Exclusively Activated by Designer Drugs (DREADDs) were developed by Roth and colleagues [20,21,32]. DREADDs are muscarinic G-protein-coupled receptors (GPCRs) that are no longer activated by acetylcholine, their endogenous ligand, but by clozapine N-oxide (CNO), an inert metabolite of the antipsychotic drug clozapine. The same actuator, CNO, can be used to either incite or silence neuronal firing through hM3Dq [7] and hM4Di [33], respectively. In the past years, new tools or improved versions have been developed [34,35], as well as new actuators such as deschloroclozapine [36,37] with better pharmacokinetics and fewer side effects. In addition, a pitfall for muscarinic-based DREADDs is that all respond to the same ligands, rendering bidirectional experiments not possible. Other chemogenetic systems responsive to different ligands have been developed to overcome this limitation in recent years. These include systems based on GPCRs like the κ-opioid receptor inhibitory DREADD named KORD and its ligand Salvinorin B (SALB) [35]. Additionally, there are ligand-gated ion channels (LGICs) like the Pharmacologically Selective Actuator Modules (PSAMs), which are engineered α7 nicotinic acetylcholine receptor (nAChR) domains that respond to small molecules termed Pharmacologically Selective Effector Molecules (PSEMs), binding to various ion-pore domains [34]. These recent additions have made it possible to control neuronal firing in the same neuronal by using solely chemogenetics.

### 2.3. Advantages and Limitations

Chemo- and optogenetic tools can be used independently and combined, as shown in many studies, being used on the same or distinct neuronal populations [38,39,40].

Despite both opto- and chemogenetics serve similar purposes, they offer different advantages and limitations that may make one or another more suitable for certain strategies and research interrogations (Table 1).

Considering the nature of the actuator, they display differences in the timing of the response and control of the stimulation. Light speed offers a high degree of temporal resolution for optogenetics as light can be easily and quickly delivered, stopped, and/or manipulated to another wavelength. In response to a precise light wavelength, a fast effect on the scale of milliseconds occurs on the targeted neuron, and the consequent physiological process in the brain can be studied [3]. On the other hand, the use of a small compound in chemogenetics does not offer the same degree of control over timing since it relies on the diffusion of the compound throughout the body, the activation of the receptor, and the clearance of the drug by the body or the *in vitro* experimental setup. Indeed, the administration of the actuator might result in a delay between the administration and the initiation of the effect, followed by several minutes to hours of effect. Despite not being able to elicit an immediate response, a sustained activation offers the possibility to study behavior and therapeutic responses that require protracted effects. In this line, optogenetics, but not chemogenetics, grants a fast-reversible effect and the possibility to better control the amount of stimulation.

As mentioned above, both techniques can be targeted to specific tissues, cell types, and/or subcellular compartments. Nevertheless, differences are observed regarding the targeted area and the invasiveness of the approach. For instance, although the coverage of the receptor extends to all the brain regions, by using optogenetics the effect will extend to the limits of light delivery. On the other hand, chemogenetics will affect a broader area and even the whole system considering the delivery approach used. Regarding the delivery approach, the small ligands used in chemogenetics can be introduced by a simple injection offering a less invasive and more flexible option. The main challenge is that the ligand should be able to cross the blood–brain barrier. Meanwhile, optogenetics requires an intracranial implant for light delivery that needs to be implanted surgically. To circumvent this limitation for the use of optogenetics, it is also possible to work with organisms whose brains are accessible by light, i.e., zebrafish larvae [41], or to use red-shifted opsins since red wavelengths pose a higher penetrance [15,42].

### 2.4. Proving Integration and Functionality

Cell therapy has been implemented in numerous animal models aiming to improve impaired function or even to replace lost cells. Undoubtedly, both optogenetics and chemogenetics have boosted the study of cellular integration and the evaluation of its benefits for the recovery of damaged neural circuits. Optogenetics is preferable when precise spatiotemporal control of a small subset of neurons is necessary, while chemogenetics offers a broad control of whole circuits of wide systems.

The rapid dynamics of the optogenetic response allows the dissection of synaptic interactions between grafted and host neurons. For instance, it is possible to express ChR2 on the host cells and perform electrophysiological recordings on the grafted cells, so afferent synapses to the grafted cells may be elucidated. Conversely, whether grafted neurons express ChR2 and recordings are performed from the host cells, efferent synapses would be detected [43,44,45]. Beyond proving functional integration, optogenetics has also been used for evaluating the improvement or recovery derived from cell therapy [46,47]. Steinbeck and colleagues used halorhodopsin (eNpHR), an inhibitory optogenetic tool, to modulate electrophysiological and neurochemical properties of human embryonic stem cell (hESC)-derived dopaminergic neurons grafted into the striatum of mice lesioned to model Parkinson’s disease. The authors proved that motor deficits were rapidly and reversibly re-introduced in recovered mice when grafted neurons were silenced by light [47]. Another example of using optogenetics to prove the direct therapeutic effect of grafted cells on the network was shown by Palma-Tortosa and colleagues, who also used halorhodopsin to silence grafted and endogenous neuronal populations and prove that functional recovery of the motor function after stroke is due to the synaptic integration of transplanted neurons [46]. Likewise, Upadhya and colleagues used the inhibitory chemogenetic tool hM4D(Gi) to prove that the reduction in seizure frequency observed after cell therapy in a model of epilepsy was due to the integration of the grafted cells [48]. In this last example, the use of chemogenetics was ideal since the effect was evaluated over a period of days.

### 2.5. Other Applications

In addition to the functional assessment of the integration, optogenetics has also been used for additional purposes related to cell therapy. For instance, by using a light-inducible system to control the expression of a transgene, i.e., Brn2, it is possible to control and understand the endogenous mechanisms governing neuronal differentiation [49]. This knowledge and system can be further used for directing the phenotype of the stem cells. In this line, Giraldo and colleagues showed that optogenetic activation of neurons could improve the survival and maturation of neural progenitor cells *in vitro* [50]. After repetitive optogenetic stimulations, authors observed an increase in the proliferation and differentiation of the grafted cells into oligodendrocytes and neurons. Moreover, neurons displayed an increased branching and axonal length. Altogether, this highlights the potential of optogenetics in improving cell therapy outcomes through improved engraftments and cellular identity, though its effectiveness remains to be proved *in vivo*.

## 3. Monitoring Neuronal Activity

For many decades, electrophysiology has been the principal method to study neuronal activity. It allows for accurately determining spike rates at high temporal resolution, although technical complexity makes it difficult to detect firings in dense neuronal populations [51]. Thereafter, optical imaging arose as a preferred approach for targeting specific cell populations to determine neuronal activity with high spatiotemporal resolution by using different sensors. Moreover, it allows to simultaneously record neuronal ensembles without damaging targeted cells [52]. The most implemented signature for functional imaging of neuronal activity has been cellular calcium, which is considered a proxy for the electrical activity of the brain. This is due to its influx in the soma and presynaptic terminals when an action potential (AP) occurs and its wide concentration changes and slow kinetics. Many different calcium indicators have been engineered throughout the years, from the use of chemical calcium indicators to the development of Genetically Encoded Calcium Indicators (GECIs) (Table 2).

The complex delivery methods required for chemical calcium indicators, alongside their limited signal-to-noise ratio, have posed significant challenges to their practical application. Consequently, GECIs have emerged as the most implemented and accurate calcium indicators. The sensor color palette of fluorescent GECIs has been widened to reduce tissue scattering, background signal, and phototoxicity as well as to increase the penetration depth, giving rise to red-shifted or near-infrared (NIR) GECIs [62]. The combination of spectrally separated GECIs allowed to perform multicolor optical imaging enabling the assessment of activity from two different cell populations implicated in a disease or behavior. This approach helps to understand spatiotemporally neuronal ensemble relationships [63,64,65,66], or even carry out paired recordings at axonal and dendritic compartments to understand the course of information through synapses [66,67]. Moreover, the use of spectrally diverse GECIs and opsins has allowed for concurrently performing calcium imaging in a particular neuronal population when optogenetically manipulating the activity of another neuronal subset. These all-optical approaches are extremely useful for elucidating causal roles of a subset of neurons within a neural network, to model circuit dysfunctions of neurodegenerative disorders (NDDs) via optogenetic manipulation while recording neighboring neurons during behavioral testing or to perform circuit mapping [68,69]. For the latter purpose, it is also important to study cell structures and functional connectivity which requires high enough basal fluorescence to clearly elucidate cell structure and subcellular compartments in both active and resting states. Some of the previously mentioned GECIs lack this capability. Therefore, aiming at developing a GECI sensor able to provide functional highlighting of both structure and neural activity, phototransformable sensors based on photoconvertion (e.g., CAMPARI) and photoactivation (e.g., Pa-G-GECO, Pa-jRCaMP, Pa-R-GECI) were engineered. Photoactivable and photoconvertible GECIs are sensors that are turned ON or whose emission spectrum changes, respectively, in an irreversible manner by near-UV irradiation [61]. Contrary to them, photoswitchable probes have recently been developed to mark and erase neurons from animals in freely moving conditions. Nevertheless, phototransformable GECI has been implemented in small animals but scarcely in rodents, giving room for further improvements to facilitate implementation in mammals [70].

### 3.1. GECIs for In Vivo Applications

Fluorescent protein-based GECIs have been the most implemented calcium indicators in neuroscience research since they allow long-term functional recordings of neuronal subsets according to cell subtype, connectivity, and circuit patterns at good signal-to-noise ratio, exhibiting high photostability and providing spike rate information [51,52]. They can even be implemented to resolve many biological questions residing in events occurring at subcellular compartments such as the soma [71] and neuronal processes including axons [72], dendrites [73], or dendritic spines [74]. Those properties make GECI systems the most suitable for the *in vivo* study of functional activity in transplanted neurons. Moreover, the possibility of generating transgenic mouse lines stably expressing GECIs [75] enables the study of neuronal dynamics along neurodevelopment or disease deficits present in NDDs [76,77,78,79,80].

### 3.2. In Vivo Calcium Imaging in Head-Restrained Animals

Optimization of fluorescent-based calcium sensors has elapsed in parallel with advancements in fluorescence microscopy for *in vivo* implementations. Although some *in vivo* calcium imaging studies have been performed using synthetic dyes [4], two-photon microscopy in combination with GCaMP family sensors has been the gold standard for monitoring and measuring *in vivo* neuronal activity in head-restrained conditions. Such implementation has provided insights into neuronal patterns and dynamics in both health and disease after cell replacement. For example, it has been implemented to unravel spatiotemporal dynamics of transplanted neural populations within the visual cortex [63,81,82]; or to study the affectations in iPSC-derived cortical neurons from Down’s syndrome patients in mice [83]. Another study has also utilized this methodology for assessing the functional integration of ESC-derived astrocytic transplants into the somatosensory cortex of adult mice [84].

Moreover, this methodology has also been used in head-restrained conditions while animals were running on a treadmill or absorbed in a virtual reality environment mimicking more unrestricted and natural conditions [85,86,87]. In addition, this setup has also been used in NDD models to elucidate pathological activity and structural signatures or study calcium dynamics in brain cells other than neurons such as microglia [88] or mural cells [89].

### 3.3. In Vivo Calcium Imaging in Freely Moving Animals

Introducing calcium imaging in awake freely behaving mice has been a challenge for many decades since head-restrained *in vivo* recordings make inaccessible the assessment of more natural behaviors with integrated environment interaction [74]. Single- and multi-photon fluorescence microscopy are difficult to implement in freely moving animals because components required for optical imaging are bulky and heavy, which has been shown to introduce bias on the behavioral performance. Moreover, head-fixed conditions involve distinct neural ensembles compared to freely navigating states [90] apart from causing stress and not considering vestibular and eye–head movement information that head restriction entails [76]. First attempts to perform *in vivo* calcium imaging in awake-behaving animals were possible with the emergence of two-photon fiber-based microscopes or “fiberscopes” [91,92]. Thereafter, miniature integrated microscopes or “miniscopes” [93] arose as a result of their miniaturized optimization to adapt it to small animals. Both technologies hold one-photon and two-photon emission options to record calcium dynamics in freely behaving conditions at cellular resolution in superficial areas by opening a cranial window or in deep regions such as the hippocampus or infralimbic cortex by implanting gradient index (GRIN) lenses or prism probes. The use of fiberscopes and miniscopes with GRIN lenses provides cellular resolution recordings of deep brain areas as well as multilayered recordings of the cortex when coupled with microprisms [94,95]. Furthermore, fiberscopes and miniscopes enable the combination of functional calcium imaging with holographic patterned optogenetic photostimulation of single and multiple neurons for all-optical interrogation of brain functions in behaving conditions [96,97,98]. Another alternative system to perform *in vivo* calcium imaging is fiber photometry, a technique that records fluorescent changes in the overall targeted neuronal population through an implanted optical cannula paired with an optical fiber that is connected to the external imaging system [99].

Each technology has its advantages and limitations; therefore, selection of imaging technology should be based on the biological question needed to be answered. Fiber photometry is the less expensive and technically demanding, quicker method that results in minimal invasiveness considering the size of the implanted fibers. It is suitable for large-scale and long-term experiments based on multiregion calcium recordings in which single-cell resolution is not required and for combining simultaneously multiregion calcium imaging with multiregion optogenetics to study natural and causal neuronal dynamics related to complex behaviors [99]. It has been mainly implemented in combination with calcium indicators to record brain areas in freely moving mice during social interaction [99,100], perform simultaneous recording of distinct neuronal populations during novel object investigation using three-color XCaMPS [66], and to elucidate the implication of neuronal subsets within a neural circuitry during specific behaviors by combining optogenetically stimulated opsins with calcium indicators [99,100,101]. The incapacity to visualize cellular structures and the limited spatial resolution in both calcium imaging and optogenetic remain still the main limitations of this method [102].

Therefore, optical fiberscopes and miniscopes constitute the preferred options to perform fine-scale *in vivo* calcium recordings at a single brain region with higher spatial resolution at the cellular and subcellular levels. Optical fiberscopes constitute the most complete technology to understand the functionality of neuronal circuits since it allows *in vivo* calcium recordings at population resolution (such as fiber photometry) as well as at single-cell resolution (such as microendoscopes). Since the development of the first two-photon fiberscopes allowing calcium imaging of multiple neurons simultaneously during *in vivo* navigating conditions [92,103], several improvements have been made to increase acquisition frequency without hampering image resolution [104], broaden the field of view and decrease motion artifacts by engineering one-photon fiberscopes [96,105], or overcome rotatory limitations generated by long optical fibers and electrical wires connected to the external device [106,107].

To overcome the later limitation, miniaturization of fiberscopes led to integrated one-photon microscopes. Portability, large FOV, and better data quality because of the all-included optical head-mounted housing avoiding long wires are some advantages of epifluorescence miniscopes compared to fiber-based microscopes. Moreover, it also reduces motion disturbances in comparison with two-photon microscopy in head-fixed mice due to increased axial resolution and faster frame rates [93]. However, clear limitations underlying one-photon miniscopes include the quality of the emission and imaging devices due to the miniaturization, the high background fluorescence arising from the lack of optical sectioning which limits depth penetration, and the necessity to develop complex algorithms to demix signals from neurons to reach single-cell resolution. Optimized wide-field miniscopes have been engineered to achieve multiplane imaging [108] or a large FOV containing several brain areas for mesoscale imaging in behaving rodents [76,78]. High-resolution two-photon miniscopes intrinsically permitting optical sectioning [74] alongside light-field based miniscopes have also been developed to achieve volumetric imaging in freely behaving mice [109,110]. Further attempts have been made to incorporate optical sectioning in one-photon miniscopes, but, to date, it has only been achieved in fixed-tissue and head-restricted *in vivo* conditions [111].

### 3.4. Other Specific Indicators of Neuronal Activity

Other indicators worth considering for monitoring neuronal activity include genetically encoded voltage indicators (GEVIs), which enable real-time reporting of the electrical activity of excitable cells on a millisecond time scale. Efforts to develop optical imaging strategies focusing on application in the mammalian brain have resulted in the development of improved GEVIs variants, including near-infrared or red-shifted GEVIs with enhanced performance properties [112,113]. Furthermore, a recent study developed a set of compatible GEVIs with opposite response polarity and colors, enabling simultaneous recording of pairs of neuronal types from the visual cortex and hippocampus with dual-polarity or dual-color voltage recordings in awake flies and mice, and up to three neuronal types by combining both recording strategies in running mice [114]. Thus, this approach constitutes a potential alternative to GECIs to perform fine-scale multipopulation recordings at millisecond resolution, in combination with one-photon and two-photon fluorescence microscopy, also reaching deep areas when used with microendoscopy approaches in mice [115].

Moreover, promising alternative indicators for future neuroscience research encompass genetically encoded fluorescence sensors for neurotransmitter dynamics. These sensors enable real-time monitoring for chemical transmission *in vivo* with high spatiotemporal precision and single-cell resolution [116]. A variety of sensors for different neurotransmitters are available, including those for glutamate, GABA, or acetylcholine, among others, and they can be detected in behaving animals by fiber photometry, stationary two-photon excitation microscopy, and miniaturized head-mounted microscopes [116,117,118].

### 3.5. Future Perspectives in Imaging Technologies for Neuronal Connectivity

Ideally, the combination of different imaging technologies will enable a more detailed and thorough insight into the functionality and connectivity of neuronal networks involved in cognitive processes, behaviors, or diseases. For example, Revah and colleagues developed an exhaustive characterization of organoid-derived grafted cells into rat somatosensory cortex by performing MRI to track grafted cells prior to calcium imaging recordings. By combining both technologies, authors studied both spontaneous and sensory-evoked activity in freely moving and anesthetized rats via fiber photometry and two-photon microscopy in head-fixed conditions, respectively [119].

The main challenges of these imaging technologies for improving their applicability are related with depth penetration into the brain and spatial and temporal resolution, but also involving analysis of tridimensional data and reducing the costs. This will require the combination of different imaging modalities, such us multiphoton microscopy, functional magnetic resonance imaging (fMRI), and positron emission tomography (PET) together with opto/chemogenetics, each offering unique strengths and capabilities for studying neuronal activity in the context of cell therapy for brain repair.

## 4. Tracers for Neuronal Functional Connectivity

**Figure 2:**
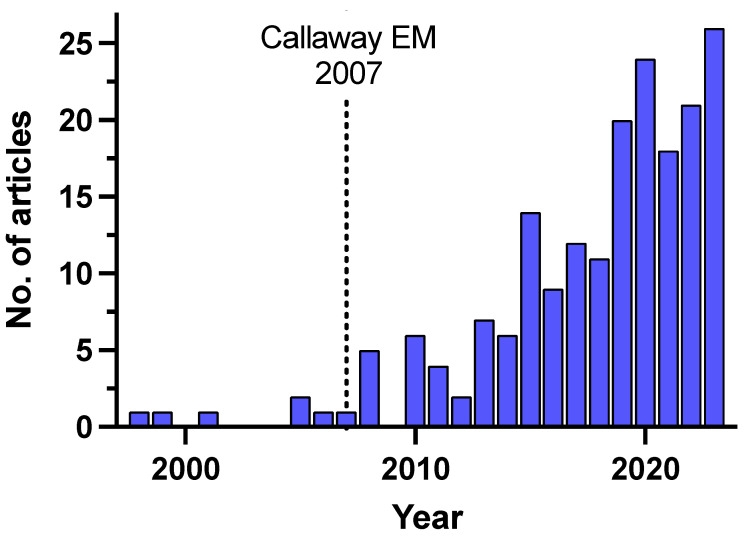
Number of articles using viral-based monosynaptic tracing. Data originated from PubMed search using the terms: “monosynaptic”, “tracing”, and “virus”. The publishing date of the paper by the laboratory of Edward M. Callaway in 2007 [122] is denoted by a dotted line.

Unraveling the intricate architecture of neural circuits is essential for understanding not only brain function and behavior but also the reconstruction of damaged neuronal networks after cell therapy. Monosynaptic tracing, a technique used to selectively label interconnected neurons, has undergone remarkable advancements with the integration of viral vectors since its first description and use in the brain [120,121] and subsequent improvement for monosynaptic specificity in 2007 [122] (Figure 2). By utilizing viral vectors with target-specific promoters, the introduced genetic material is expressed only in the desired neuron population, allowing the virus to spread exclusively to connected neurons. This provides valuable insights into how transplanted cells interact with existing neural networks and offers unique information about how cell therapy impacts neural circuitry and contributes to functional recovery [1].

**Figure 3:**
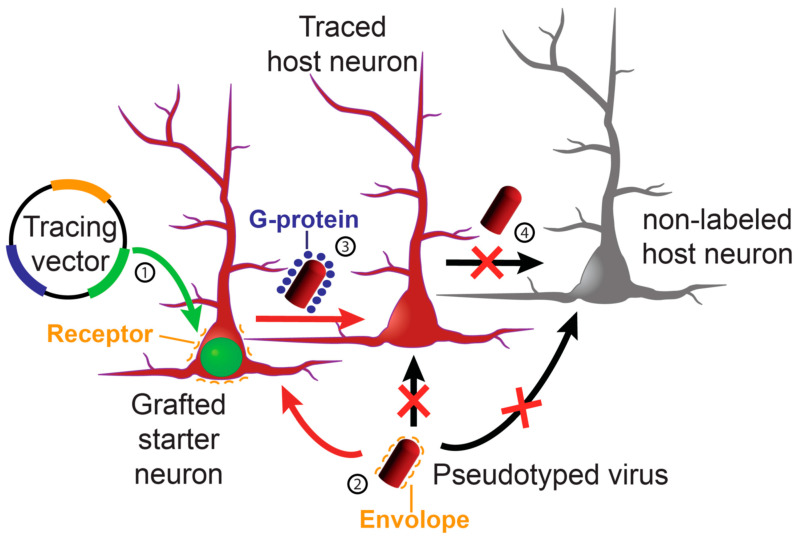
Schematic representation of the process for monosynaptic tracing using viral vectors. The references to the numbers (from 1 to 4) are explained in the text (Adapted from [123]).

The process for monosynaptic tracing using viral vectors involves several steps [122], as illustrated in Figure 3. First, the so-called “tracing vector” is introduced into the cells (1) that will receive the original infection with the tracing virus, those are the “starter” neurons. This vector will include a reporter molecule (green in the example, i.e., GFP), a receptor for the specific infection (orange), and the protein needed for the synaptic transmission of the virus (G-protein, purple). Second, the starter neurons will be infected with the pseudotyped virus (2), which also contains a reporter gene (red in the example, i.e., mCherry), thanks to the presence of a specific envelope recognized by the receptor (orange). And third, the presence of the protein for synaptic transmission (G-protein introduced by the tracing vector) will allow the pseudotyped virus to infect cells that are connected to them by functional synapses (3), spreading the reporter (red in the example) to those “traced” neurons. Since the “traced” neurons do not express the protein for synaptic transmission (4), the tracing will be restricted to monosynaptically connected neurons.

Among the various methods available for monosynaptic tracing, the use of viruses has gained significant attention due to several important advantages. The precision of viral targeting enables researchers to selectively label specific neurons and their connected partners, minimizing noise and enhancing the accuracy of circuit reconstruction. Additionally, viruses can be engineered to express fluorescent proteins or other markers, enabling visualization of labeled neurons in exquisite detail. This capability facilitates comprehensive mapping of neural pathways, shedding light on complex circuit architectures with unprecedented clarity. Finally, the flexibility of viral vectors also allows for cell-type-specific targeting, enabling researchers to dissect subpopulations within a given neural network, including tracing from individual neurons [124].

### 4.1. Retrograde Tracing Using Rabies Virus

Rabies virus was the first viral vector extensively used for monosynaptic tracing and has retrograde preference [125]. Its ability to propagate across synapses in a retrograde manner allows researchers to label neurons that provide inputs to a targeted region [126]. By engineering the rabies virus to express fluorescent markers, such as GFP or tdTomato, researchers can track the source of inputs and reconstruct the upstream neural network. This technique has been pivotal in unraveling intricate neural pathways in various brain regions and has provided valuable insights into sensory, motor, and cognitive circuits in animal models [127].

Regarding cell therapy studies, rabies virus-based monosynaptic tracing has been crucial for addressing functional integration of transplanted cells in several animal models such as hESCs-derived cells injected into the striatum [127], hiPSC-derived cells in the cortex after ischemic stroke [46,123], or mouse embryonic cells after neuronal ablation in the visual cortex [82,128,129], among others. In addition, this method has been used to confirm afferent and efferent synaptic connections between human cortical organotypic cultures and hiPSC-derived grafted neurons [130], the first human-to-human relevant *ex vivo* model for cell therapy described in the literature.

### 4.2. Anterograde Tracing Using Herpes Virus

Herpes simplex virus (HSV) has been harnessed for anterograde monosynaptic tracing, allowing the visualization of axonal projections from a given neuron. In particular, HSV1 strain H1129 has shown predominant anterograde transneuronal transmission [131]. By engineering HSV to express fluorescent markers, axons of a specific neuron and their projections to downstream targets can be labeled. Recently, a novel monosynaptic anterograde tracing system based on the deletion of the gene UL6 from the genome of a Cre-dependent version of the anterograde Herpes Simplex Virus 1 strain H129 has been described [132], providing a reliable method to label postsynaptic partners.

These anterograde tracing techniques have been particularly useful in elucidating neural connectivity patterns in both the central and peripheral nervous systems [133] and provide a unique tool for the study of functional integration of grafted cells in cell therapy approaches.

### 4.3. Future Perspectives in Mapping Communication Pathways after Cell Transplantation

Monosynaptic tracing tools employing viruses have revolutionized our understanding of neural circuitry. These techniques offer unparalleled specificity, enabling researchers to uncover intricate connections and communication pathways within the nervous system [5]. The use of rabies virus for retrograde labeling and herpes virus for anterograde tracing has provided invaluable insights into various brain regions and functions. As technology continues to advance, viral monosynaptic tracing tools are poised to play an increasingly pivotal role in unraveling the complex architecture of the brain.

The challenges of monosynaptic tracing technology include improvements in efficiency and cell specificity that can arrive to a single starting neuron [122,124]; the combination of tracing with electrophysiology in acute slices, optogenetics, and chemogenetics that allows the study and modulation of neuronal activity specifically in traced neurons [134,135]; and the generation of detailed functional connectivity maps by combining it with spatial omics technologies, not only in healthy brain but also in diseased environment [136], for a better understanding of neurological and psychiatric disorders.

## 5. Conclusions

Currently, several relatively new technologies are used alone or in combination allowing a deep exploration of functional integration and circuit reconstruction after cell transplantation. Altogether, continuous optimization and broadening of both microscopical techniques and indicators of neuronal activity as well as their increased affordability in the neuroscience field would enable multiplexed implementations of such techniques for a more exhaustive characterization of neuronal circuits to understand functional integration of grafted neurons.

Better knowledge of the functional integration processes of transplanted cells and, therefore, the mechanisms that direct network repair, will lead to the development of new therapeutic strategies or the improvement of existing ones. So, despite certain limitations and technical complexity, these techniques contribute to the refinement and validation of cell therapy, bringing it closer to clinical application. Furthermore, some of them represent a therapeutic strategy by themselves, such as the use of opto/chemogenetics as an alternative to electrical brain stimulation [137,138,139].

## Figures and Tables

**Table 1:** Advantages and limitations of optogenetics and chemogenetics.

	Optogenetics	Chemogenetics
**Timing of the response**	Fast (milliseconds)	Prolonged, rely on pharmacokinetics
**Control of stimulation**	Exogenous	Endogenous
**Target area**	Restricted to the illuminated area	Engineered cells in all the body
**Invasiveness**	Invasive	Noninvasive
**Examples**		
Excitation	ChR2, ChETA, ChrimsonR	hM3Dq, PSAM4-5HT3
Inhibition	Halo, eNpHR3.0, ArchT	hM4Di, KORD, PSAM4-GlyR

**Table 2:** Chemical and Genetically Encoded Calcium Indicators.

Calcium Sensor	Category	Indicators	Description	Ref.
**Chemical calcium indicators**	Synthetic ratiometric	Fura-2	High-affinity Ca^2+^ indicator that shifts its excitation wavelength depending on whether Ca^2+^ is bound.	[53,54]
	Single-wavelength	Fluo-4	High-affinity Ca^2+^ indicator that boosts fluorescence upon Ca^2+^ binding.	
**Genetically encoded calcium indicators** (**GECIs**)	Fluorescence resonance energy transfer (FRET)-based	Cameleon	ECFP and EYFP are fused to calmodulin (CaM) and calmodulin-target peptide (M13), resulting in an increase in the FRET signal upon Ca^2+^ binding.	[55,56]
	Single fluorescent protein (FP)-based	Camgaroo-1	CaM is fused to YFP, thus allowing Ca^2+^ to induce an increase in fluorescence by causing a conformational change in CaM.	[52,57]
		Pericams	Circularly permuted YFP (cpYFP) is fused to CaM and M13. The binding of calcium allows CaM/M13 to fold tightly against YFP, greatly increasing fluorescence.	[58,59]
		GCaMP family	Circularly permuted GFP is fused to CaM and M13. Many different GCaMP variants have been designed (from GCaMP1 to GCaMP8).	[60,61]

## Data Availability

Not applicable.

## References

[1] Tornero D. (2022). Neuronal Circuitry Reconstruction after Stem Cell Therapy in Damaged Brain. Neural Regen. Res..

[2] Palma-Tortosa S., Coll-San Martin B., Kokaia Z., Tornero D. (2021). Neuronal Replacement in Stem Cell Therapy for Stroke: Filling the Gap. Front. Cell Dev. Biol..

[3] Deisseroth K. (2015). Optogenetics: 10 Years of Microbial Opsins in Neuroscience. Nat. Neurosci..

[4] Stosiek C., Garaschuk O., Holthoff K., Konnerth A. (2003). In Vivo Two-Photon Calcium Imaging of Neuronal Networks. Proc. Natl. Acad. Sci. USA.

[5] Kim E.J., Jacobs M.W., Ito-Cole T., Callaway E.M. (2016). Improved Monosynaptic Neural Circuit Tracing Using Engineered Rabies Virus Glycoproteins. Cell Rep..

[6] Garner A.R., Rowland D.C., Hwang S.Y., Baumgaertel K., Roth B.L., Kentros C., Mayford M. (2012). Generation of a Synthetic Memory Trace. Science.

[7] Alexander G.M., Rogan S.C., Abbas A.I., Armbruster B.N., Pei Y., Allen J.A., Nonneman R.J., Hartmann J., Moy S.S., Nicolelis M.A. (2009). Remote Control of Neuronal Activity in Transgenic Mice Expressing Evolved G Protein-Coupled Receptors. Neuron.

[8] Teissier A., Chemiakine A., Inbar B., Bagchi S., Ray R.S., Palmiter R.D., Dymecki S.M., Moore H., Ansorge M.S. (2015). Activity of Raphé Serotonergic Neurons Controls Emotional Behaviors. Cell Rep..

[9] Boyden E.S., Zhang F., Bamberg E., Nagel G., Deisseroth K. (2005). Millisecond-Timescale, Genetically Targeted Optical Control of Neural Activity. Nat. Neurosci..

[10] Zhang F., Wang L.-P., Brauner M., Liewald J.F., Kay K., Watzke N., Wood P.G., Bamberg E., Nagel G., Gottschalk A. (2007). Multimodal Fast Optical Interrogation of Neural Circuitry. Nature.

[11] Nagel G., Szellas T., Huhn W., Kateriya S., Adeishvili N., Berthold P., Ollig D., Hegemann P., Bamberg E. (2003). Channelrhodopsin-2, a Directly Light-Gated Cation-Selective Membrane Channel. Proc. Natl. Acad. Sci. USA.

[12] Nagel G., Ollig D., Fuhrmann M., Kateriya S., Musti A.M., Bamberg E., Hegemann P. (2002). Channelrhodopsin-1: A Light-Gated Proton Channel in Green Algae. Science.

[13] Chow B.Y., Han X., Dobry A.S., Qian X., Chuong A.S., Li M., Henninger M.A., Belfort G.M., Lin Y., Monahan P.E. (2010). High-Performance Genetically Targetable Optical Neural Silencing by Light-Driven Proton Pumps. Nature.

[14] Sengupta A., Chaffiol A., Macé E., Caplette R., Desrosiers M., Lampič M., Forster V., Marre O., Lin J.Y., Sahel J. (2016). Red-shifted Channelrhodopsin Stimulation Restores Light Responses in Blind Mice, Macaque Retina, and Human Retina. EMBO Mol. Med..

[15] Gong X., Mendoza-Halliday D., Ting J.T., Kaiser T., Sun X., Bastos A.M., Wimmer R.D., Guo B., Chen Q., Zhou Y. (2020). An Ultra-Sensitive Step-Function Opsin for Minimally Invasive Optogenetic Stimulation in Mice and Macaques. Neuron.

[16] Gunaydin L.A., Yizhar O., Berndt A., Sohal V.S., Deisseroth K., Hegemann P. (2010). Ultrafast Optogenetic Control. Nat. Neurosci..

[17] Baker C.K., Flannery J.G. (2018). Innovative Optogenetic Strategies for Vision Restoration. Front. Cell Neurosci..

[18] Zhang F., Aravanis A.M., Adamantidis A., de Lecea L., Deisseroth K. (2007). Circuit-Breakers: Optical Technologies for Probing Neural Signals and Systems. Nat. Rev. Neurosci..

[19] Lin J.Y. (2011). A User’s Guide to Channelrhodopsin Variants: Features, Limitations and Future Developments. Exp. Physiol..

[20] Roth B.L. (2016). DREADDs for Neuroscientists. Neuron.

[21] Dong S., Rogan S.C., Roth B.L. (2010). Directed Molecular Evolution of DREADDs: A Generic Approach to Creating next-Generation RASSLs. Nat. Protoc..

[22] Strader C.D., Gaffney T., Sugg E.E., Candelore M.R., Keys R., Patchett A.A., Dixon R.A. (1991). Allele-Specific Activation of Genetically Engineered Receptors. J. Biol. Chem..

[23] Magnus C.J., Lee P.H., Atasoy D., Su H.H., Looger L.L., Sternson S.M. (2011). Chemical and Genetic Engineering of Selective Ion Channel–Ligand Interactions. Science.

[24] Arenkiel B.R., Klein M.E., Davison I.G., Katz L.C., Ehlers M.D. (2008). Genetic Control of Neuronal Activity in Mice Conditionally Expressing TRPV1. Nat. Methods.

[25] Klein G., Humbert N., Gradinaru J., Ivanova A., Gilardoni F., Rusbandi U.E., Ward T.R. (2005). Tailoring the Active Site of Chemzymes by Using a Chemogenetic-Optimization Procedure: Towards Substrate-Specific Artificial Hydrogenases Based on the Biotin-Avidin Technology. Angew. Chem. Int. Ed..

[26] Collot J., Gradinaru J., Humbert N., Skander M., Zocchi A., Ward T.R. (2003). Artificial Metalloenzymes for Enantioselective Catalysis Based on Biotin−Avidin. J. Am. Chem. Soc..

[27] Dar A.C., Das T.K., Shokat K.M., Cagan R.L. (2012). Chemical Genetic Discovery of Targets and Anti-Targets for Cancer Polypharmacology. Nature.

[28] Cohen M.S., Zhang C., Shokat K.M., Taunton J. (2005). Structural Bioinformatics-Based Design of Selective, Irreversible Kinase Inhibitors. Science.

[29] Bishop A.C., Shah K., Liu Y., Witucki L., Kung C., Shokat K.M. (1998). Design of Allele-Specific Inhibitors to Probe Protein Kinase Signaling. Curr. Biol..

[30] Conklin B.R., Hsiao E.C., Claeysen S., Dumuis A., Srinivasan S., Forsayeth J.R., Guettier J.-M., Chang W.C., Pei Y., McCarthy K.D. (2008). Engineering GPCR Signaling Pathways with RASSLs. Nat. Methods.

[31] Pei Y., Rogan S.C., Yan F., Roth B.L. (2008). Engineered GPCRs as Tools to Modulate Signal Transduction. Physiology.

[32] Pei Y., Dong S., Roth B.L. (2010). Generation of Designer Receptors Exclusively Activated by Designer Drugs (DREADDs) Using Directed Molecular Evolution. Curr. Protoc. Neurosci..

[33] Armbruster B.N., Li X., Pausch M.H., Herlitze S., Roth B.L. (2007). Evolving the Lock to Fit the Key to Create a Family of G Protein-Coupled Receptors Potently Activated by an Inert Ligand. Proc. Natl. Acad. Sci. USA.

[34] Magnus C.J., Lee P.H., Bonaventura J., Zemla R., Gomez J.L., Ramirez M.H., Hu X., Galvan A., Basu J., Michaelides M. (2019). Ultrapotent Chemogenetics for Research and Potential Clinical Applications. Science.

[35] Vardy E., Robinson J.E., Li C., Olsen R.H.J., DiBerto J.F., Giguere P.M., Sassano F.M., Huang X.-P., Zhu H., Urban D.J. (2015). A New DREADD Facilitates the Multiplexed Chemogenetic Interrogation of Behavior. Neuron.

[36] Nagai Y., Miyakawa N., Takuwa H., Hori Y., Oyama K., Ji B., Takahashi M., Huang X.-P., Slocum S.T., DiBerto J.F. (2020). Deschloroclozapine, a Potent and Selective Chemogenetic Actuator Enables Rapid Neuronal and Behavioral Modulations in Mice and Monkeys. Nat. Neurosci..

[37] Chen X., Choo H., Huang X.-P., Yang X., Stone O., Roth B.L., Jin J. (2015). The First Structure–Activity Relationship Studies for Designer Receptors Exclusively Activated by Designer Drugs. ACS Chem. Neurosci..

[38] Cai H., Haubensak W., Anthony T.E., Anderson D.J. (2014). Central Amygdala PKC-Δ+ Neurons Mediate the Influence of Multiple Anorexigenic Signals. Nat. Neurosci..

[39] Miao C., Cao Q., Ito H.T., Yamahachi H., Witter M.P., Moser M.-B., Moser E.I. (2015). Hippocampal Remapping after Partial Inactivation of the Medial Entorhinal Cortex. Neuron.

[40] Betley J.N., Xu S., Cao Z.F.H., Gong R., Magnus C.J., Yu Y., Sternson S.M. (2015). Neurons for Hunger and Thirst Transmit a Negative-Valence Teaching Signal. Nature.

[41] Portugues R., Severi K.E., Wyart C., Ahrens M.B. (2013). Optogenetics in a Transparent Animal: Circuit Function in the Larval Zebrafish. Curr. Opin. Neurobiol..

[42] Chuong A.S., Miri M.L., Busskamp V., Matthews G.A.C., Acker L.C., Sørensen A.T., Young A., Klapoetke N.C., Henninger M.A., Kodandaramaiah S.B. (2014). Noninvasive Optical Inhibition with a Red-Shifted Microbial Rhodopsin. Nat. Neurosci..

[43] Cunningham M., Cho J.-H., Leung A., Savvidis G., Ahn S., Moon M., Lee P.K.J., Han J.J., Azimi N., Kim K.-S. (2014). HPSC-Derived Maturing GABAergic Interneurons Ameliorate Seizures and Abnormal Behavior in Epileptic Mice. Cell Stem Cell.

[44] Waloschková E., Gonzalez-Ramos A., Mikroulis A., Kudláček J., Andersson M., Ledri M., Kokaia M. (2021). Human Stem Cell-Derived GABAergic Interneurons Establish Efferent Synapses onto Host Neurons in Rat Epileptic Hippocampus and Inhibit Spontaneous Recurrent Seizures. Int. J. Mol. Sci..

[45] Gonzalez-Ramos A., Waloschková E., Mikroulis A., Kokaia Z., Bengzon J., Ledri M., Andersson M., Kokaia M. (2021). Human Stem Cell-Derived GABAergic Neurons Functionally Integrate into Human Neuronal Networks. Sci. Rep..

[46] Palma-Tortosa S., Tornero D., Hansen M.G., Monni E., Hajy M., Kartsivadze S., Aktay S., Tsupykov O., Parmar M., Deisseroth K. (2020). Activity in Grafted Human IPS Cell–Derived Cortical Neurons Integrated in Stroke-Injured Rat Brain Regulates Motor Behavior. Proc. Natl. Acad. Sci. USA.

[47] Steinbeck J.A., Choi S.J., Mrejeru A., Ganat Y., Deisseroth K., Sulzer D., Mosharov E.V., Studer L. (2015). Optogenetics Enables Functional Analysis of Human Embryonic Stem Cell-Derived Grafts in a Parkinson’s Disease Model. Nat. Biotechnol..

[48] Upadhya D., Hattiangady B., Castro O.W., Shuai B., Kodali M., Attaluri S., Bates A., Dong Y., Zhang S.-C., Prockop D.J. (2019). Human Induced Pluripotent Stem Cell-Derived MGE Cell Grafting after Status Epilepticus Attenuates Chronic Epilepsy and Comorbidities via Synaptic Integration. Proc. Natl. Acad. Sci. USA.

[49] Sokolik C., Liu Y., Bauer D., McPherson J., Broeker M., Heimberg G., Qi L.S., Sivak D.A., Thomson M. (2015). Transcription Factor Competition Allows Embryonic Stem Cells to Distinguish Authentic Signals from Noise. Cell Syst..

[50] Giraldo E., Palmero-Canton D., Martinez-Rojas B., Sanchez-Martin M.D.M., Moreno-Manzano V. (2020). Optogenetic Modulation of Neural Progenitor Cells Improves Neuroregenerative Potential. Int. J. Mol. Sci..

[51] Inoue M. (2021). Genetically Encoded Calcium Indicators to Probe Complex Brain Circuit Dynamics In Vivo. Neurosci. Res..

[52] Lin M.Z., Schnitzer M.J. (2016). Genetically Encoded Indicators of Neuronal Activity. Nat. Neurosci..

[53] Tsien R.Y. (1980). New Calcium Indicators and Buffers with High Selectivity against Magnesium and Protons: Design, Synthesis, and Properties of Prototype Structures. Biochemistry.

[54] Paredes R.M., Etzler J.C., Watts L.T., Zheng W., Lechleiter J.D. (2008). Chemical Calcium Indicators. Methods.

[55] Miyawaki A., Llopis J., Heim R., McCaffery J.M., Adams J.A., Ikura M., Tsien R.Y. (1997). Fluorescent Indicators for Ca^2+^ based on Green Fluorescent Proteins and Calmodulin. Nature.

[56] Pérez Koldenkova V., Nagai T. (2013). Genetically Encoded Ca^2+^ Indicators: Properties and Evaluation. Biochim. Biophys. Acta Mol. Cell Res..

[57] Baird G.S., Zacharias D.A., Tsien R.Y. (1999). Circular Permutation and Receptor Insertion within Green Fluorescent Proteins. Proc. Natl. Acad. Sci. USA.

[58] Nagai T., Sawano A., Park E.S., Miyawaki A. (2001). Circularly Permuted Green Fluorescent Proteins Engineered to Sense Ca^2+^. Proc. Natl. Acad. Sci. USA.

[59] Wu N., Nishioka W.K., Derecki N.C., Maher M.P. (2019). High-Throughput-Compatible Assays Using a Genetically-Encoded Calcium Indicator. Sci. Rep..

[60] Nakai J., Ohkura M., Imoto K. (2001). A High Signal-to-Noise Ca2+ Probe Composed of a Single Green Fluorescent Protein. Nat. Biotechnol..

[61] Heinrich R., Hussein W., Berlin S. (2021). Photo-Transformable Genetically-Encoded Optical Probes for Functional Highlighting In Vivo. J. Neurosci. Methods.

[62] Hashizume R., Fujii H., Mehta S., Ota K., Qian Y., Zhu W., Drobizhev M., Nasu Y., Zhang J., Bito H. (2022). A Genetically Encoded Far-red Fluorescent Calcium Ion Biosensor Derived from a Biliverdin-binding Protein. Protein Sci..

[63] Chen T.-W., Wardill T.J., Sun Y., Pulver S.R., Renninger S.L., Baohan A., Schreiter E.R., Kerr R.A., Orger M.B., Jayaraman V. (2013). Ultrasensitive Fluorescent Proteins for Imaging Neuronal Activity. Nature.

[64] Dana H., Mohar B., Sun Y., Narayan S., Gordus A., Hasseman J.P., Tsegaye G., Holt G.T., Hu A., Walpita D. (2016). Sensitive Red Protein Calcium Indicators for Imaging Neural Activity. eLife.

[65] Sonoda K., Matsui T., Bito H., Ohki K. (2018). Astrocytes in the Mouse Visual Cortex Reliably Respond to Visual Stimulation. Biochem. Biophys. Res. Commun..

[66] Inoue M., Takeuchi A., Manita S., Horigane S., Sakamoto M., Kawakami R., Yamaguchi K., Otomo K., Yokoyama H., Kim R. (2019). Rational Engineering of XCaMPs, a Multicolor GECI Suite for In Vivo Imaging of Complex Brain Circuit Dynamics. Cell.

[67] Yang W., Yuste R. (2017). In Vivo Imaging of Neural Activity. Nat. Methods.

[68] Resta F., Montagni E., de Vito G., Scaglione A., Allegra Mascaro A.L., Pavone F.S. (2022). Large-Scale All-Optical Dissection of Motor Cortex Connectivity Shows a Segregated Organization of Mouse Forelimb Representations. Cell Rep..

[69] Carrillo-Reid L., Han S., Yang W., Akrouh A., Yuste R. (2019). Controlling Visually Guided Behavior by Holographic Recalling of Cortical Ensembles. Cell.

[70] Sha F., Abdelfattah A.S., Patel R., Schreiter E.R. (2020). Erasable Labeling of Neuronal Activity Using a Reversible Calcium Marker. eLife.

[71] Chen Y., Jang H., Spratt P.W.E., Kosar S., Taylor D.E., Essner R.A., Bai L., Leib D.E., Kuo T.-W., Lin Y.-C. (2020). Soma-Targeted Imaging of Neural Circuits by Ribosome Tethering. Neuron.

[72] Broussard G.J., Liang Y., Fridman M., Unger E.K., Meng G., Xiao X., Ji N., Petreanu L., Tian L. (2018). In Vivo Measurement of Afferent Activity with Axon-Specific Calcium Imaging. Nat. Neurosci..

[73] Tang Y., Li L., Sun L., Yu J., Hu Z., Lian K., Cao G., Dai J. (2020). In Vivo Two-Photon Calcium Imaging in Dendrites of Rabies Virus-Labeled V1 Corticothalamic Neurons. Neurosci. Bull..

[74] Zong W., Wu R., Li M., Hu Y., Li Y., Li J., Rong H., Wu H., Xu Y., Lu Y. (2017). Fast High-Resolution Miniature Two-Photon Microscopy for Brain Imaging in Freely Behaving Mice. Nat. Methods.

[75] Dana H., Chen T.-W., Hu A., Shields B.C., Guo C., Looger L.L., Kim D.S., Svoboda K. (2014). Thy1-GCaMP6 Transgenic Mice for Neuronal Population Imaging In Vivo. PLoS ONE.

[76] Rynes M.L., Surinach D.A., Linn S., Laroque M., Rajendran V., Dominguez J., Hadjistamoulou O., Navabi Z.S., Ghanbari L., Johnson G.W. (2021). Miniaturized Head-Mounted Microscope for Whole-Cortex Mesoscale Imaging in Freely Behaving Mice. Nat. Methods.

[77] Cramer J.V., Gesierich B., Roth S., Dichgans M., Düring M., Liesz A. (2019). In Vivo Widefield Calcium Imaging of the Mouse Cortex for Analysis of Network Connectivity in Health and Brain Disease. Neuroimage.

[78] Scott B.B., Thiberge S.Y., Guo C., Tervo D.G.R., Brody C.D., Karpova A.Y., Tank D.W. (2018). Imaging Cortical Dynamics in GCaMP Transgenic Rats with a Head-Mounted Widefield Macroscope. Neuron.

[79] Bai Y., Li M., Zhou Y., Ma L., Qiao Q., Hu W., Li W., Wills Z.P., Gan W.-B. (2017). Abnormal Dendritic Calcium Activity and Synaptic Depotentiation Occur Early in a Mouse Model of Alzheimer’s Disease. Mol. Neurodegener..

[80] Calvo-Rodriguez M., Kharitonova E.K., Bacskai B.J. (2021). In Vivo Brain Imaging of Mitochondrial Ca^2+^ in Neurodegenerative Diseases with Multiphoton Microscopy. Biochim. Biophys. Acta Mol. Cell Res..

[81] Linaro D., Vermaercke B., Iwata R., Ramaswamy A., Libé-Philippot B., Boubakar L., Davis B.A., Wierda K., Davie K., Poovathingal S. (2019). Xenotransplanted Human Cortical Neurons Reveal Species-Specific Development and Functional Integration into Mouse Visual Circuits. Neuron.

[82] Falkner S., Grade S., Dimou L., Conzelmann K.K., Bonhoeffer T., Götz M., Hübener M. (2016). Transplanted Embryonic Neurons Integrate into Adult Neocortical Circuits. Nature.

[83] Real R., Peter M., Trabalza A., Khan S., Smith M.A., Dopp J., Barnes S.J., Momoh A., Strano A., Volpi E. (2018). In Vivo Modeling of Human Neuron Dynamics and Down Syndrome. Science.

[84] Zhang K., Chen C., Yang Z., He W., Liao X., Ma Q., Deng P., Lu J., Li J., Wang M. (2016). Sensory Response of Transplanted Astrocytes in Adult Mammalian Cortex In Vivo. Cereb. Cortex.

[85] Cichon J., Gan W.-B. (2015). Branch-Specific Dendritic Ca^2+^ Spikes Cause Persistent Synaptic Plasticity. Nature.

[86] Dombeck D.A., Harvey C.D., Tian L., Looger L.L., Tank D.W. (2010). Functional Imaging of Hippocampal Place Cells at Cellular Resolution during Virtual Navigation. Nat. Neurosci..

[87] Morcos A.S., Harvey C.D. (2016). History-Dependent Variability in Population Dynamics during Evidence Accumulation in Cortex. Nat. Neurosci..

[88] Umpierre A.D., Bystrom L.L., Ying Y., Liu Y.U., Worrell G., Wu L.-J. (2020). Microglial Calcium Signaling Is Attuned to Neuronal Activity in Awake Mice. eLife.

[89] Tong L., Hill R.A., Damisah E.C., Murray K.N., Yuan P., Bordey A., Grutzendler J. (2021). Imaging and Optogenetic Modulation of Vascular Mural Cells in the Live Brain. Nat. Protoc..

[90] Werner C.T., Williams C.J., Fermelia M.R., Lin D.-T., Li Y. (2019). Circuit Mechanisms of Neurodegenerative Diseases: A New Frontier With Miniature Fluorescence Microscopy. Front. Neurosci..

[91] Helmchen F., Fee M.S., Tank D.W., Denk W. (2001). A Miniature Head-Mounted Two-Photon Microscope. Neuron.

[92] Myaing M.T., MacDonald D.J., Li X. (2006). Fiber-Optic Scanning Two-Photon Fluorescence Endoscope. Opt. Lett..

[93] Ghosh K.K., Burns L.D., Cocker E.D., Nimmerjahn A., Ziv Y., El Gamal A., Schnitzer M.J. (2011). Miniaturized Integration of a Fluorescence Microscope. Nat. Methods.

[94] Ziv Y., Ghosh K.K. (2015). Miniature Microscopes for Large-Scale Imaging of Neuronal Activity in Freely Behaving Rodents. Curr. Opin. Neurobiol..

[95] Cameron C.M., Murugan M., Choi J.Y., Engel E.A., Witten I.B. (2019). Increased Cocaine Motivation Is Associated with Degraded Spatial and Temporal Representations in IL-NAc Neurons. Neuron.

[96] Szabo V., Ventalon C., De Sars V., Bradley J., Emiliani V. (2014). Spatially Selective Holographic Photoactivation and Functional Fluorescence Imaging in Freely Behaving Mice with a Fiberscope. Neuron.

[97] Accanto N., Blot F.G.C., Lorca-Cámara A., Zampini V., Bui F., Tourain C., Badt N., Katz O., Emiliani V. (2023). A Flexible Two-Photon Fiberscope for Fast Activity Imaging and Precise Optogenetic Photostimulation of Neurons in Freely Moving Mice. Neuron.

[98] Zhang J., Hughes R.N., Kim N., Fallon I.P., Bakhurin K., Kim J., Severino F.P.U., Yin H.H. (2022). A One-Photon Endoscope for Simultaneous Patterned Optogenetic Stimulation and Calcium Imaging in Freely Behaving Mice. Nat. Biomed. Eng..

[99] Gunaydin L.A., Grosenick L., Finkelstein J.C., Kauvar I.V., Fenno L.E., Adhikari A., Lammel S., Mirzabekov J.J., Airan R.D., Zalocusky K.A. (2014). Natural Neural Projection Dynamics Underlying Social Behavior. Cell.

[100] Kim C.K., Yang S.J., Pichamoorthy N., Young N.P., Kauvar I., Jennings J.H., Lerner T.N., Berndt A., Lee S.Y., Ramakrishnan C. (2016). Simultaneous Fast Measurement of Circuit Dynamics at Multiple Sites across the Mammalian Brain. Nat. Methods.

[101] Nieh E.H., Vander Weele C.M., Matthews G.A., Presbrey K.N., Wichmann R., Leppla C.A., Izadmehr E.M., Tye K.M. (2016). Inhibitory Input from the Lateral Hypothalamus to the Ventral Tegmental Area Disinhibits Dopamine Neurons and Promotes Behavioral Activation. Neuron.

[102] Legaria A.A., Matikainen-Ankney B.A., Yang B., Ahanonu B., Licholai J.A., Parker J.G., Kravitz A.V. (2022). Fiber Photometry in Striatum Reflects Primarily Nonsomatic Changes in Calcium. Nat. Neurosci..

[103] Sawinski J., Wallace D.J., Greenberg D.S., Grossmann S., Denk W., Kerr J.N.D. (2009). Visually Evoked Activity in Cortical Cells Imaged in Freely Moving Animals. Proc. Natl. Acad. Sci. USA.

[104] Guan H., Li D., Park H., Li A., Yue Y., Gau Y.-T.A., Li M.-J., Bergles D.E., Lu H., Li X. (2022). Deep-Learning Two-Photon Fiberscopy for Video-Rate Brain Imaging in Freely-Behaving Mice. Nat. Commun..

[105] Flusberg B.A., Nimmerjahn A., Cocker E.D., Mukamel E.A., Barretto R.P.J., Ko T.H., Burns L.D., Jung J.C., Schnitzer M.J. (2008). High-Speed, Miniaturized Fluorescence Microscopy in Freely Moving Mice. Nat. Methods.

[106] Li A., Guan H., Park H.-C., Yue Y., Chen D., Liang W., Li M.-J., Lu H., Li X. (2021). Twist-Free Ultralight Two-Photon Fiberscope Enabling Neuroimaging on Freely Rotating/Walking Mice. Optica.

[107] Gengatharan A., Malvaut S., Marymonchyk A., Ghareghani M., Snapyan M., Fischer-Sternjak J., Ninkovic J., Götz M., Saghatelyan A. (2021). Adult Neural Stem Cell Activation in Mice Is Regulated by the Day/Night Cycle and Intracellular Calcium Dynamics. Cell.

[108] Barbera G., Liang B., Zhang L., Li Y., Lin D.-T. (2019). A Wireless MiniScope for Deep Brain Imaging in Freely Moving Mice. J. Neurosci. Methods.

[109] Skocek O., Nöbauer T., Weilguny L., Martínez Traub F., Xia C.N., Molodtsov M.I., Grama A., Yamagata M., Aharoni D., Cox D.D. (2018). High-Speed Volumetric Imaging of Neuronal Activity in Freely Moving Rodents. Nat. Methods.

[110] Zong W., Wu R., Chen S., Wu J., Wang H., Zhao Z., Chen G., Tu R., Wu D., Hu Y. (2021). Miniature Two-Photon Microscopy for Enlarged Field-of-View, Multi-Plane and Long-Term Brain Imaging. Nat. Methods.

[111] Supekar O.D., Sias A., Hansen S.R., Martinez G., Peet G.C., Peng X., Bright V.M., Hughes E.G., Restrepo D., Shepherd D.P. (2022). Miniature Structured Illumination Microscope for in Vivo 3D Imaging of Brain Structures with Optical Sectioning. Biomed. Opt. Express.

[112] Song C., Matlashov M.E., Shcherbakova D.M., Antic S.D., Verkhusha V.V., Knöpfel T. (2023). Characterization of Two Near-Infrared Genetically Encoded Voltage Indicators. Neurophotonics.

[113] Han Y., Yang J., Li Y., Chen Y., Ren H., Ding R., Qian W., Ren K., Xie B., Deng M. (2023). Bright and Sensitive Red Voltage Indicators for Imaging Action Potentials in Brain Slices and Pancreatic Islets. Sci. Adv..

[114] Kannan M., Vasan G., Haziza S., Huang C., Chrapkiewicz R., Luo J., Cardin J.A., Schnitzer M.J., Pieribone V.A. (2022). Dual-Polarity Voltage Imaging of the Concurrent Dynamics of Multiple Neuron Types. Science.

[115] Davis H.C., III F.P.B., Wong-Campos J.D., Cohen A.E. (2023). Optical Constraints on Two-Photon Voltage Imaging. bioRxiv.

[116] Leopold A.V., Shcherbakova D.M., Verkhusha V.V. (2019). Fluorescent Biosensors for Neurotransmission and Neuromodulation: Engineering and Applications. Front. Cell Neurosci..

[117] Marvin J.S., Scholl B., Wilson D.E., Podgorski K., Kazemipour A., Müller J.A., Schoch S., Quiroz F.J.U., Rebola N., Bao H. (2018). Stability, Affinity, and Chromatic Variants of the Glutamate Sensor IGluSnFR. Nat. Methods.

[118] Marvin J.S., Shimoda Y., Magloire V., Leite M., Kawashima T., Jensen T.P., Kolb I., Knott E.L., Novak O., Podgorski K. (2019). A Genetically Encoded Fluorescent Sensor for in Vivo Imaging of GABA. Nat. Methods.

[119] Revah O., Gore F., Kelley K.W., Andersen J., Sakai N., Chen X., Li M.-Y., Birey F., Yang X., Saw N.L. (2022). Maturation and Circuit Integration of Transplanted Human Cortical Organoids. Nature.

[120] Card J., Rinaman L., Lynn R., Lee B., Meade R., Miselis R., Enquist L. (1993). Pseudorabies Virus Infection of the Rat Central Nervous System: Ultrastructural Characterization of Viral Replication, Transport, and Pathogenesis. J. Neurosci..

[121] Lee H.S., Lee I.S., Kang T.-C., Seo J., Lee B.H. (1998). The Distribution of Calcitonin Gene-Related Peptide in Gastric Vagal Circuit of Rats. Anat. Histol. Embryol..

[122] Wickersham I.R., Lyon D.C., Barnard R.J.O., Mori T., Finke S., Conzelmann K.K., Young J.A.T., Callaway E.M. (2007). Monosynaptic Restriction of Transsynaptic Tracing from Single, Genetically Targeted Neurons. Neuron.

[123] Tornero D., Tsupykov O., Granmo M., Rodriguez C., Grønning-Hansen M., Thelin J., Smozhanik E., Laterza C., Wattananit S., Ge R. (2017). Synaptic Inputs from Stroke-Injured Brain to Grafted Human Stem Cell-Derived Neurons Activated by Sensory Stimuli. Brain.

[124] Masaki Y., Yamaguchi M., Takeuchi R.F., Osakada F. (2022). Monosynaptic Rabies Virus Tracing from Projection-Targeted Single Neurons. Neurosci. Res..

[125] Lin K.-Z., Li L., Ma W.-Y., Yang X., Han Z.-P., Luo N.-S., Wang J., Xu F.-Q. (2022). A Rabies Virus–Based Toolkit for Efficient Retrograde Labeling and Monosynaptic Tracing. Neural Regen. Res..

[126] Callaway E.M., Luo L. (2015). Monosynaptic Circuit Tracing with Glycoprotein-Deleted Rabies Viruses. J. Neurosci..

[127] Grealish S., Heuer A., Cardoso T., Kirkeby A., Jönsson M., Johansson J., Björklund A., Jakobsson J., Parmar M. (2015). Monosynaptic Tracing Using Modified Rabies Virus Reveals Early and Extensive Circuit Integration of Human Embryonic Stem Cell-Derived Neurons. Stem Cell Rep..

[128] Grade S., Thomas J., Zarb Y., Thorwirth M., Conzelmann K.-K., Hauck S.M., Götz M. (2022). Brain Injury Environment Critically Influences the Connectivity of Transplanted Neurons. Sci. Adv..

[129] Thomas J., Fernanda Martinez-Reza M., Thorwirth M., Zarb Y., Conzelmann K.-K., Hauck S.M., Grade S., Götz M. (2022). Excessive Local Host-Graft Connectivity in Aging and Amyloid-Loaded Brain. Sci. Adv..

[130] Grønning Hansen M., Laterza C., Palma-Tortosa S., Kvist G., Monni E., Tsupykov O., Tornero D., Uoshima N., Soriano J., Bengzon J. (2020). Grafted Human Pluripotent Stem Cell-Derived Cortical Neurons Integrate into Adult Human Cortical Neural Circuitry. Stem Cells Transl. Med..

[131] Yang H., Xiong F., Qin H.B., Yu Q.T., Sun J.Y., Zhao H.W., Li D., Zhou Y., Zhang F.K., Zhu X.W. (2022). A Novel H129-Based Anterograde Monosynaptic Tracer Exhibits Features of Strong Labeling Intensity, High Tracing Efficiency, and Reduced Retrograde Labeling. Mol. Neurodegener..

[132] Fischer K.B., Collins H.K., Pang Y., Roy D.S., Zhang Y., Feng G., Li S., Kepecs A., Callaway E.M. (2023). Monosynaptic Restriction of the Anterograde Herpes Simplex Virus Strain H129 for Neural Circuit Tracing. J. Comp. Neurol..

[133] Xiong F., Yang H., Song Y.G., Qin H.B., Zhang Q.Y., Huang X., Jing W., Deng M., Liu Y., Liu Z. (2022). An HSV-1-H129 Amplicon Tracer System for Rapid and Efficient Monosynaptic Anterograde Neural Circuit Tracing. Nat. Commun..

[134] Vivar C., Potter M.C., Choi J., Lee J., Stringer T.P., Callaway E.M., Gage F.H., Suh H., van Praag H. (2012). Monosynaptic Inputs to New Neurons in the Dentate Gyrus. Nat. Commun..

[135] Kato S., Kobayashi K. (2020). Pseudotyped Lentiviral Vectors for Tract-Targeting and Application for the Functional Control of Selective Neural Circuits. J. Neurosci. Methods.

[136] Rodríguez-Urgellés E., Casas-Torremocha D., Sancho-Balsells A., Ballasch I., García-García E., Miquel-Rio L., Manasanch A., del Castillo I., Chen W., Pupak A. (2023). Thalamic Foxp2 Regulates Output Connectivity and Sensory-Motor Impairments in a Model of Huntington’s Disease. Cell. Mol. Life Sci..

[137] Miyakawa N., Nagai Y., Hori Y., Mimura K., Orihara A., Oyama K., Matsuo T., Inoue K., Suzuki T., Hirabayashi T. (2023). Chemogenetic Attenuation of Cortical Seizures in Nonhuman Primates. Nat. Commun..

[138] Sahel J.-A., Boulanger-Scemama E., Pagot C., Arleo A., Galluppi F., Martel J.N., Esposti S.D., Delaux A., de Saint Aubert J.-B., de Montleau C. (2021). Partial Recovery of Visual Function in a Blind Patient after Optogenetic Therapy. Nat. Med..

[139] Bryson J.B., Kourgiantaki A., Jiang D., Demosthenous A., Greensmith L. (2024). An Optogenetic Cell Therapy to Restore Control of Target Muscles in an Aggressive Mouse Model of Amyotrophic Lateral Sclerosis. eLife.

